# Tuberculose multifocale associée à une toxoplasmose cérébrale sur terrain immunodéprimé à VIH chez un patient immigré africain au Centre Hospitalier de Soissons, France

**DOI:** 10.11604/pamj.2018.31.47.15916

**Published:** 2018-09-20

**Authors:** Mamadou Oury Safiatou Diallo, Moussa Sidibé, Ibrahima Bah, Fode Bangaly Sako, Karamba Sylla, Fode Amara Traoré, Aminata Oumou Sylla, Mamadou Saliou Sow, Ali Abdessand Hachémi, François Boiquigny, Mohamed Cisse

**Affiliations:** 1 Service des Maladies Infectieuses de l’Hôpital National Donka, Université Gamal Abdel Nasser de Conakry, Conakry, République de Guinée; 2 Service d’Imagerie Médicale de l’Hôpital Militaire d’Abidjan, Côte d’Ivoire; 3 Service de Médecine Interne du Centre Hospitalier de Soissons, Avenue Du General De Gaulle, Soissons, France; 4 Service d’Imagerie Médicale du Centre Hospitalier de Soissons, Avenue Du General De Gaulle, Soissons, France; 5 Service de Dermatologie de l’Hôpital National Donka, Université Gamal Abdel Nasser de Conakry, Conakry, République de Guinée

**Keywords:** Tuberculose, toxoplasmose, VIH, immigré, Soissons, France, Tuberculosis, toxoplasmosis, HIV, immigrant, Soissons, France

## Abstract

Dans les pays industrialisés et notamment en France, vu les moyens de prévention, dépistage précoce et prise en charge immédiate de l'infection à VIH, la survenue d'infections opportunistes ne se voit presque chez les immigrés et certaines couches socio-professionnelles défavorisées. Nous rapportons donc le cas d'un homme de 42 ans, immigré africain, hospitalisé pour syndrome infectieux dans un contexte d'altération de l'état général, VIH1 positif sous antirétroviraux depuis deux ans, arrêtés depuis quatre mois, une tuberculose pulmonaire traitée et déclarée guérie en février 2017 avec, à l'examen une lenteur à l'idéation, une fièvre à 39,6°C et un amaigrissement. Le nadir CD4 à 12/mm^3^, une charge virale VIH1 à 5,80log. Le scanner thoraco-abdominal et l'IRM cérébrale ont permis de visualiser des lésions intra-abdomino-thoraciques et cérébrales avant la confirmation diagnostique de la tuberculose et de la toxoplasmose. Le patient fut mis sous trithérapie antirétrovirale à quinze jours du traitement antituberculeux, puis au traitement antitoxoplasmique avec une évolution favorable.

## Abstract (en)

In the industrialized countries and, in particular, in France, given the means of prevention, early screening and treatment of HIV infection, there is almost no evidence of opportunistic infections among immigrants and in some disadvantaged socio-professional groups. We here report the case of a 42-year old African immigrant HIV1 positive man with impaired general condition hospitalized for infectious syndrome. He had received antiretroviral therapy for 2 years and stopped it four months before admission. He had had pulmonary tuberculosis treated and declared cured in February 2017. Clinical examination showed slow ideation, a temperature of 39.6°C and weight loss. The patient had nadir CD4 cell counts 12/mm3 and HIV viral load log value 5.80. Thoracoabdominal CT scan and brain MRI showed intra-abdominal and thoracic lesions as well as brain lesions before diagnostic confirmation of tuberculosis and toxoplasmosis. The patient underwent triple antiretroviral therapy on day 15 of antituberculosis treatment. Then he underwent toxoplasmosis treatment with favorable outcome.

## Introduction

Le stade le plus avancé de l'infection à VIH est le syndrome d'immunodéficience acquise (SIDA), qui peut mettre 2 à 15 ans selon le cas. Ce stade se définit par l'apparition de certains cancers, infections ou d'autres manifestations cliniques [1, 2]. Le niveau d'immunodépression conditionne le risque de survenue et le type de manifestations opportunistes. Ces infections ou tumeurs opportunistes peuvent survenir simultanément chez un patient ou se succéder dans le temps en cas de persistance du déficit immunitaire [2]. Le risque de développer une tuberculose est multiplié par sept en cas d'infection par le VIH. En France, l'incidence de la co-infection est particulièrement élevée chez les patients originaires d'Afrique subsaharienne et d'Europe centrale. Elle est fréquemment révélatrice de l'infection VIH et est actuellement parmi les infections opportunistes les plus fréquentes [2]. La toxoplasmose cérébrale, infection parasitaire, réactivation d'une infection ancienne, est la plus fréquente des infections opportunistes du système nerveux central en France. Elle survient tardivement au cours de l'évolution du SIDA notamment chez les patients ne recevant pas de prophylaxie. La toxoplasmose cérébrale consiste en des abcès cérébraux généralement multiples. Elle réalise un tableau neurologique focal dans environ la moitié des cas avec déficit [2].

## Patient et observation

Un patient de 42 ans d'origine africaine en situation d'asile politique en France depuis Mars 2017, transféré d'une structure sanitaire pour prise en charge d'un syndrome infectieux et d'une altération de l'état général évoluant depuis une quinzaine de jours environ. La sérologie VIH1 positive depuis trois ans sous ARV (Truvada+Effavirenz), inobservant avec interruption du traitement depuis quatre mois (mars à juillet 2017), aux antécédents de tuberculose pulmonaire en 2016, traitée et déclarée guérie en février 2017 et notion de tuberculose pulmonaire chez son épouse et chez ses deux enfants tous traités et déclarés guéris en Aout 2016. L'examen clinique retrouvait un patient asthénique avec un amaigrissement non chiffré, fébrile au toucher, conjonctives bien colorées, anictériques, pas d'œdème aux membres inférieurs. Les constantes sont: température: 39,2°C, TA: 100/60 mmHg, IMC: 16,2kg/m^2^. Sur le plan neurologique un ralentissement idéo-moteur, répondant péniblement à nos questions, sans syndrome méningé, ni signes de focalisation, reflexes conservés. Aux poumons, pas de signes fonctionnels respiratoires, murmure vésiculaire positif aux deux champs, pas de râles. Au plan digestif, pas d'enduit blanchâtre buccal, bon état bucco-dentaire, pas de troubles digestifs (douleur, diarrhée, vomissement), pas de sensibilité abdominale. On note une tachycardie régulière sans bruit surajouté. Pas de lésions cutanées, pas de signes fonctionnels urinaires.

**Figure 1:**
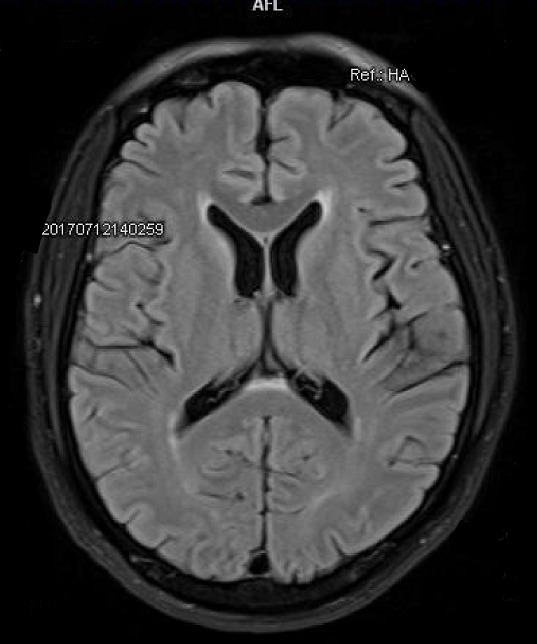
Imagerie par résonnance magnétique normale

**Figure 2:**
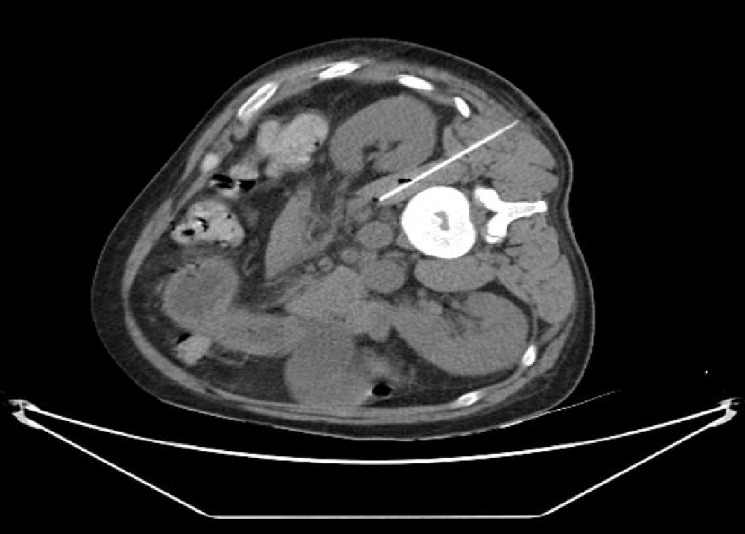
Ponction sous scannographique des adénopathies intra-abdominales

La biologie du 12 Juillet 2017 met en évidence: GB: 6.2giga/l, Hb: 11.7g/dl, Plaquettes: 181giga/l, cytolyse et cholestase hépatiques, créatininémie normale à 79 micromol/l, CRP: 307mg/l, sérologie toxoplasmique: IgG et IgM positives, toxo latex: positif au 1/32, sérologies VHB, VHC, VHE, CMV, SYPHYLIS, LYME: négatives. L'IRM réalisée le 12 /07/2017 était sans particularités (Figure 1). La fibroscopie oesogastro-duodénale réalisée le 12 Juillet 2017: normale. Ponction lombaire faite: LCR clair eau de roche, 0 élément, stérile à la culture. Un scanner thoraco-abdomino-pelvien réalisé le 13 Juillet 2017 dans le cadre d'un bilan infectieux retrouve: des adénopathies retro-péritonéales à centre nécrotique; une hépato-splénomégalie hétérogène et un micronodule du lobe supérieur du poumon droit. Ponction des adénopathies intra-abdominales s/scannographique (Figure 2) et tubage gastrique le matin au réveil à la recherche de bacille de Koch (BK). Résultat: isolement du bacille de BK dans les deux prélèvements et présence d'ADN de mycobactéries du complexe tuberculosis à la PCR Temps Réel, l'antibiogramme du complexe tuberculosis a montré la sensibilité du bacille à la rifampicine, l'isoniazide, la pyrazynamide et l'ethambutol. La sérologie VIH est revenue positive au type 1 avec une charge virale de 5,24log (171965 copies/ml). Le génotypage montre une résistance du VIH au Saquinavir/ritonavir, à tous les inhibiteurs de la réverse transcriptase (sauf la stavudine), au tenofovir et sensible à tous les anti-intégrases. La recherche de l'allèle HLA B57,01 est revenue négative. Sur le plan immunologique: TCD4 à 12/mm^3^ (3,2%), TCD8 à 174/mm^3^ (44,6%), rapport CD4/CD8 à 0,07.

**Figure 3:**
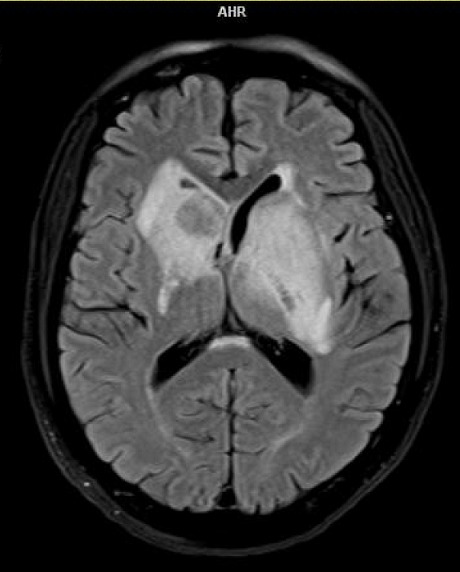
Deux (2) lésions nodulaires avec rehaussement périphérique et une zone de nécrose centrale, se situant dans la région des noyaux gris centraux à l’imagerie par résonnance magnétique

**Figure 4:**
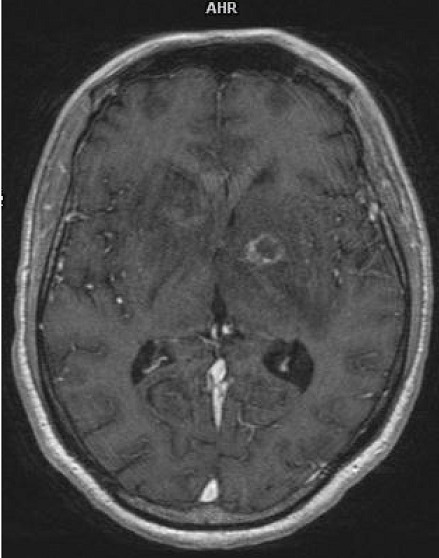
Diminution de la taille des abcès toxoplasmiques intracérébraux

L'IRM cérébrale refaite le 08 Aout 2017 devant la persistance de la lenteur idéo-motrice a mis en évidence la présence de deux (2) lésions nodulaires avec rehaussement périphérique et une zone de nécrose centrale, se situant dans la région des noyaux gris centraux faisant évoquer en première intention la toxoplasmose cérébrale (Figure 3). Présence d'ADN Toxoplasma gondii dans le LCR de la deuxième ponction lombaire par PCR. Le diagnostic de tuberculose multifocale et toxoplasmose cérébrale sur terrain d'immunodépression à VIH stade IV de l'OMS a été retenu. Nous avons procédé à une déclaration du cas à l'agence régionale de santé et à l'isolement respiratoire du patient jusqu'à la négativation du bacille de Koch sur les prélèvements respiratoires. Sur le plan thérapeutique, notre patient a été mis sous sulfamétoxazol triméthoprime pour la prévention primaire de la toxoplasmose et de la pneumocystose, puis sous antituberculeux à base de Rifabutine-Isoniazide-Pyrazynamide-Ethambutol à jeun le matin et quinze jours après, le traitement antirétroviral à base de Tenofovir-darunavir/ritonavir-raltégravir débuté. En fin l'association de sulfadiazine et Pyriméthamine+acide folinique a été mise en place pour le traitement curatif de la toxoplasmose cérébrale. L'évolution a été favorable sur la plan clinique (apyréxie, nette régression de la lenteur à l'idéation,…) et paraclinique (négativation du bacille de Koch dans les prélèvements respiratoires, diminution de la taille des abcès toxoplasmiques intracérébraux (Figure 4), disparition des adénopathies intra-abdominales, normalisation de la taille du foie et de la rate, baisse de la charge virale).

## Discussion

L'expression clinique de la tuberculose chez le VIH positif est remarquable par la diffusion des lésions avec dissémination des bacilles dans les sites autres que pulmonaire. Souvent associées à une atteinte pulmonaire, les localisations ganglionnaires périphériques et/ou profondes, les atteintes séreuses, surtout pleurale, mais aussi péritonéale, péricardique et méningée sont les plus fréquentes. La présentation clinique des tuberculoses extrapulmonaires est souvent trompeuse, ce qui entraine un retard diagnostique [3]. La tomodensitométrie thoracique, réservée aux formes complexes dont l'image ne peut être correctement interprétée sur les clichés standards et l'imagerie extrapulmonaire, indispensable pour évoquer le diagnostic et le retentissement des formes extrapulmonaires [4]. L'anatomo-pathologie: la découverte d'un granulome épithélioide et giganto-cellulaire oriente vers le diagnostic de tuberculose [4]. Le diagnostic bactériologique consiste à la mise en évidence du caractère acido-alcoolo-résistant des mycobactéries par les colorations de Zielh-neelsen ou par l'auramie, puis en culture sur milieux solide (Lowenstein-Jensen) et liquide, ensuite la détection directe de *Mycobactérium tuberculosis* par PCR permet de différencier rapidement *M.tuberculosis* d'une autre mycobactérie. Un antibiogramme systématique est réalisé pour l'isoniazide, strepomycine, éthambutol, pyrazynamide et rifampicine [4].

La présentation clinique de la toxoplasmose cérébrale est variable d'un patient à l'autre. Les patients présentent le plus souvent un syndrome d'hypertension intracrânienne d'installation rapidement progressive (céphalées, troubles de vigilance, nausées, vomissements, paralysie de la 4^ème^ paire crânienne uni ou bilatérale), des signes neurologiques focaux (59%) et/ou des signes encéphalitiques non systématisés (ralentissement psychomoteur, syndrome confusionnel) pouvant aller jusqu'au coma [5]. Le diagnostic, suspecté devant toute anomalie neurologique centrale chez une personne séropositive pour le VIH, doit faire pratiquer en urgence un examen neuroradiologique (TDM ou IRM) qui apporte des arguments morphologiques [2]. Le diagnostic biologique est direct: mise en évidence des toxoplasmes par les techniques de coloration (Giemsa, Hémalun-Eosine) et d'immunomarquage (immunofluorescence directe et immunopéroxydase) permettent l'identification directe du parasite sur fragments biopsiques et liquides biologiques (moelle, liquide de lavage broncho-alvéolaires, épanchements divers, LCR,…) lorsque la charge parasitaire est élevée [6, 7]. Le principe du traitement antituberculeux est d'associer plusieurs antituberculeux, afin d'avoir une action complémentaire sur les différentes populations de Bacilles de Koch et d'éviter la sélection de mutants résistants et la persistance de bacilles à métabolisme lent. Le traitement comprend: l'administration quotidienne en une prise orale d'isoniazide à la dose de 4-5 mg/kg/j et de rifampicine à la dose de 10 mg/kg/j. Pendant les deux premiers mois du traitement, il y est adjoint de pyrazynamide, à la dose de 20-25mg/kg/j et de l'éthambutol, à la dose de 15-20 mg/kg/j. La durée totale du traitement est de six mois dans la grande majorité des cas, y compris pour la plupart des formes extrapulmonaires (ganglionnaires, osseuses, séreuses, uro-génitales) et chez les immunodéprimés (dont le VIH). La vitamine B6 est prescrite pour la prévention systématique de la neuropathie périphérique causée par l'isoniazide chez les patients à risque (grossesse, alcoolisme, dénutrition, neuropathie préexistante, insuffisance rénale, infection par le VIH). Des compléments alimentaires: en cas de dénutrition [4, 8].

Le traitement de la toxoplasmose cérébrale repose sur l'association de pyriméthamine (100 mg à j1, puis 1mg/kg/j soit 50-75mg/j associée à 25mg d'acide folinique) et sulfadiazine (100mg/kg/j en 4 prises avec un maximum de 6g/j, auquel il faut associer une alcalinisation des urines pour éviter les lithiases) pendant au moins 6 semaines et jusqu'à réponse clinique et radiologique. En cas d'intolérance aux sulfamides, l'alternative à la sulfadiazine est la clindamycine (2.4 g/j en 4 administrations sous forme intraveineuse ou orale) [9]. Pour un premier traitement antirétroviral, il convient de recourir à une association de trois antirétroviraux actifs (trithérapie), en faisant appel à l'un des schémas suivants: 2INTI+IP; 2INTI+1INNTI ou 2INTI+INI. Il est recommandé de réaliser un test génotypique de résistance lors du diagnostic de l'infection par le VIH et de choisir le traitement en tenant compte de ces données [2]. Le premier traitement antirétroviral doit permettre de rendre la charge virale indétectable en six mois. Au cours des premiers mois de traitement, il convient de réaliser une mesure de la charge virale plasmatique: A M1, date à laquelle la charge virale plasmatique (CV) doit avoir baissé de 2 log; A M3, date à laquelle la charge virale doit être inférieure à 400 copies/ml; A M6, date à laquelle la CV doit être inférieure à 50 copies/ml. Chez certaines PVVIH, ces objectifs ne sont pas atteints et la charge virale ne devient indétectable qu'après plus de six mois de traitement [10].

## Conclusion

*Les infections opportunistes (toxoplasmose cérébrale, tuberculose,…)* au cours du sida sont parfois d'orientation voire de confirmation diagnostique difficile et ne s'observent qu'exclusivement chez des groupes de patients défavorisés dans les pays industrialisés d'où l'utilisation des moyens d'exploration les plus sensibles.

## Conflits d’intérêts

Les auteurs ne déclarent aucun conflit d'intérêts.
